# Proteome of monocyte priming by lipopolysaccharide, including changes in interleukin-1beta and leukocyte elastase inhibitor

**DOI:** 10.1186/1477-5956-6-13

**Published:** 2008-05-20

**Authors:** Michael J Pabst, Karen M Pabst, David B Handsman, Sarka Beranova-Giorgianni, Francesco Giorgianni

**Affiliations:** 1 Dental Research Center and Department of Periodontology, College of Dentistry, University of Tennessee Health Science Center, Memphis, TN 38163, USA; 2 Department of Pharmaceutical Sciences, College of Pharmacy, University of Tennessee Health Science Center, Memphis, TN 38163, USA; 3 Department of Neurology, and the Charles B. Stout Mass Spectrometry Laboratory, College of Medicine, University of Tennessee Health Science Center, Memphis, TN 38163, USA

## Abstract

**Background:**

Monocytes can be primed in vitro by lipopolysaccharide (LPS) for release of cytokines, for enhanced killing of cancer cells, and for enhanced release of microbicidal oxygen radicals like superoxide and peroxide. We investigated the proteins involved in regulating priming, using 2D gel proteomics.

**Results:**

Monocytes from 4 normal donors were cultured for 16 h in chemically defined medium in Teflon bags ± LPS and ± 4-(2-aminoethyl)-benzenesulfonyl fluoride (AEBSF), a serine protease inhibitor. LPS-primed monocytes released inflammatory cytokines, and produced increased amounts of superoxide. AEBSF blocked priming for enhanced superoxide, but did not affect cytokine release, showing that AEBSF was not toxic. After staining large-format 2D gels with Sypro ruby, we compared the monocyte proteome under the four conditions for each donor. We found 30 protein spots that differed significantly in response to LPS or AEBSF, and these proteins were identified by ion trap mass spectrometry.

**Conclusion:**

We identified 19 separate proteins that changed in response to LPS or AEBSF, including ATP synthase, coagulation factor XIII, ferritin, coronin, HN ribonuclear proteins, integrin alpha IIb, pyruvate kinase, ras suppressor protein, superoxide dismutase, transketolase, tropomyosin, vimentin, and others. Interestingly, in response to LPS, precursor proteins for interleukin-1β appeared; and in response to AEBSF, there was an increase in elastase inhibitor. The increase in elastase inhibitor provides support for our hypothesis that priming requires an endogenous serine protease.

## Background

Priming of monocytes in vitro with LPS is a model for macrophage activation, a key process in innate immunity. Innate immunity protects us against infection before specific antibodies or specific T-cell responses can be mounted. In experimental models of infection, activating macrophages in animals with agents like muramyl dipeptide cures the animals of an otherwise lethal infection [1,2]. The goal of this study was to better understand monocyte activation by LPS. So, we examined the monocyte proteome ± LPS ± AEBSF (4-(2-aminoethyl)-benzenesulfonyl fluoride), a serine protease inhibitor and an inhibitor of priming for enhanced superoxide release [3]).

In our model of macrophage activation, we cultured freshly isolated monocytes from normal adult blood donors at 37° in 5% CO_2 _in chemically defined medium (modified Earle's balanced salt solution, EBSS). Defined medium and other precautions were designed to avoid inadvertent contamination by bacterial products. All reagents and equipment were tested for microbial contamination by *Limulus *assay. To avoid artefacts caused by adherence to foreign surfaces, the monocytes were cultured in suspension in Teflon bags. We used a highly purified *E. coli *LPS preparation that is free of contaminating proteins [4], and that was active at a concentration of 2 ng/ml. (Actually, macrophages require only 1 to 10 molecules of this LPS per monocyte to become primed for enhanced release of superoxide [5]. This is a remarkable example of amplification in signal transduction in which 10 molecules of LPS enable the release of 10 billion molecules of superoxide. This is also a reminder of the likely range of concentrations of proteins involved in priming.)

In this model, we investigated the specific effects of adding LPS to monocytes in vitro. We expected fewer changes in the proteome compared with experimental systems that involve more complex biological changes, such as activation of macrophages in vivo by infection, or differentiation of monocytes into dendritic cells [6] or alveolar macrophages [7], or differentiation of a leukemic cell line [8]. Because we were looking at a small specific step (addition of LPS in vitro), we expected that changes seen in the proteome would have a high probability of being related to LPS priming.

Our model has a weakness in that we must wait some period of time (24–48 h) for freshly isolated monocytes to become fully quiescent with respect to priming for enhanced phorbol myristate acetate-triggered release of superoxide. Thus, we are mixing the effects of priming in the LPS-treated cells with the process of quiescence in the control cells. In our model, we added LPS at the beginning of culture, although we could have waited until the cells were quiescent before adding LPS. However, pure LPS, at low concentrations (2 ng/ml), takes at least 6–12 h to prime monocytes, extending the incubation time to 30–60 h. After 30–60 h, unprimed non-adherent and non-contaminated monocytes begin to undergo apoptosis, so we preferred mixing priming with quiescence rather than with apoptosis. We believe our model is a reasonable choice, but other models of macrophage and neutrophil activation have their own advantages. For example, neutrophils respond much faster to priming by LPS, and they don't require a period of incubation to achieve quiescence [9]. Monocytes respond more quickly to cytokines than to LPS [10,11].

Earlier work led us to form the hypothesis that priming of monocytes by LPS for enhanced release of microbicidal oxygen radicals like superoxide and peroxide was regulated by a negative regulator protein that normally kept the monocytes in check. Thus, any sort of infection, inflammation, or environmental stimulus leads to the activation of a protease that cleaves the negative regulator and allows the monocytes to produce large amounts of oxygen radicals. We found that bacterial products like LPS or muramyl dipeptide, cytokines like interferon-gamma, environmental challenges like gamma radiation, low pH, or hypertonicity, all prime monocytes for enhanced release of superoxide [12-14]. Our earlier work showed that macrophages could be primed by exposure to the serine proteases elastase and cathepsin G from neutrophils [15]. Priming by serine proteases, and blockade of priming by AEBSF [3], led us to suggest that proteolytic cleavage by a serine protease is part of the mechanism of monocyte priming for enhanced superoxide release (although not required for cytokine release). We hoped this proteomic study would show us the negative regulator, the protease, or some related protein. In fact, we found that LPS caused an increase in intracellular precursors for IL-1β, although earlier we showed that secretion of cytokines was not required for priming for superoxide [16]. We also found an increased amount of elastase inhibitor, when monocytes were treated with AEBSF. We suggest the hypothesis that AEBSF prevents the consumption of elastase inhibitor by inactivating the protease (elastase or some other serine protease) that regulates priming for superoxide. Of course, AEBSF inhibition of other serine proteases that are not related to priming might also contribute to the increase in elastase inhibitor.

## Results

### Cytokine release

**Figure 1:**
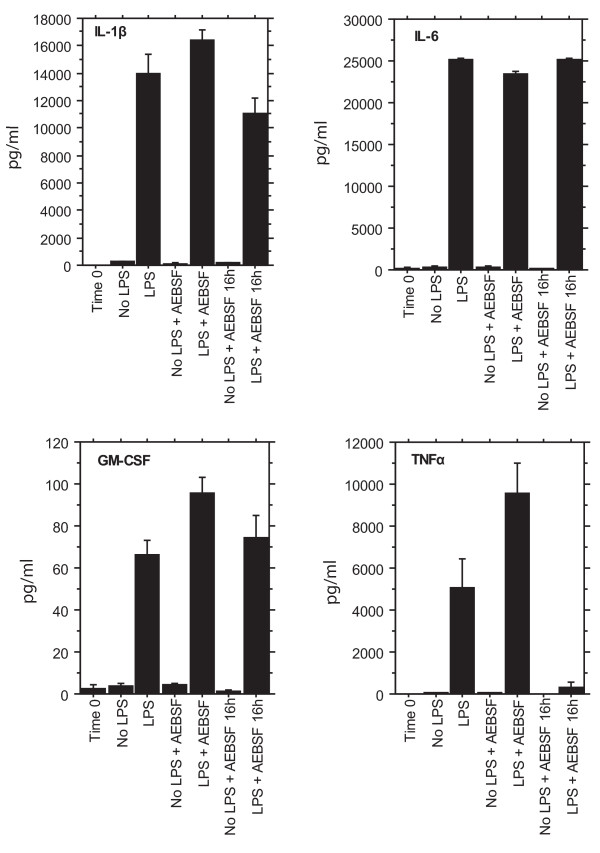
**Effects of LPS and AEBSF on cytokine secretion by monocytes**. Four of the ten cytokines assayed in culture medium from monocytes cultured for 16 h ± LPS ± AEBSF are shown. The first bar is Time 0, a control of monocyte medium taken at the start of culture. The last two bars show the effect of AEBSF added at the end of the culture at 16 h. Results are means ± SE, n = 4 donors. LPS was required for cytokine expression; AEBSF did not interfere, except that AEBSF added at the end of the experiment did reduce TNFα secretion. IL-8 and IL-10 responses were similar to IL-1β and IL-6 (not shown).

LPS-primed monocytes released inflammatory cytokines, including interleukin-1beta (IL-1β), IL-6, IL-8, IL-10, tumor necrosis factor alpha (TNFα), and granulocyte/macrophage colony stimulating factor (GM-CSF) (Figure 1). Taking IL-1β as an example, monocytes produced almost no IL-β in the absence of LPS (< 0.2 ng/ml of culture medium), whereas LPS-primed monocytes secreted 14 ± 1.5 ng/ml (mean ± SE, n = 4, P < 0.001 by ANOVA). Cytokines produced by lymphocytes, such as interferon gamma, were not found, showing that the monocyte populations were reasonably pure. AEBSF did not affect cytokine release, showing that AEBSF did not adversely affect the viability, or the protein synthesis and secretion mechanisms of the monocytes. (An anomaly was that AEBSF added at the end of the experiment, but not at the beginning, did reduce TNFα.) In earlier work, we showed that LPS-induced secretion of cytokines could be blocked by corticosteroids like dexamethasone [16].

### Superoxide release

**Figure 2:**
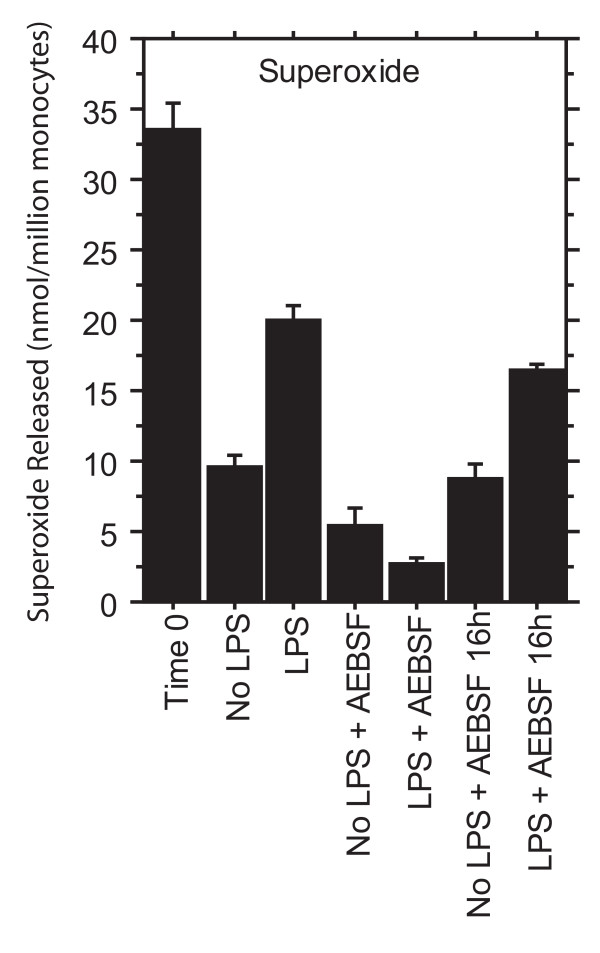
**Effects of LPS and AEBSF on phorbol-triggered superoxide release**. Phorbol myristate acetate-triggered release of superoxide from monocytes cultured for 16 h ± LPS ± AEBSF. The first bar is Time 0, a control of monocytes at the start of culture when they were still agitated by the isolation procedure. The last two bars show the effect of AEBSF added at the end of the culture at 16 h. LPS primed monocytes for enhanced release of superoxide; AEBSF blocked priming. Means ± SE, n = 4.

The success of LPS priming was also measured by determining the increase in phorbol myristate acetate-triggered release of superoxide (Figure 2). Monocytes incubated for 16 h without LPS produced 9.6 ± 0.9 nmol of superoxide per million cells, whereas monocytes primed with LPS produced 20.0 ± 1.0 nmol (mean ± SE, n = 4, P < 0.001 by ANOVA). (Even under these endotoxin-free, non-adherent culture conditions, monocytes become primed by the trauma of the isolation procedure, so the unprimed cells still produced considerable superoxide after 16 h. In our earlier paper [17], we incubated the cells for 48 h, allowing the unprimed monocytes to become more quiescent. Here we wished to capture earlier events in priming, so we accepted a less dramatic difference between unprimed and primed monocytes in superoxide release.)

AEBSF blocked priming for enhanced superoxide release. When AEBSF was added to unprimed monocytes, superoxide release was reduced to 5.5 ± 1.5 nmol; and when AEBSF was added to LPS-primed monocytes, superoxide was reduced to 2.7 ± 0.3 nmol (P < 0.001 for the effects of both LPS and AEBSF by ANOVA). AEBSF had only a small effect when added at the end of the 16 h incubation with LPS (16.6 ± 0.3 nmol versus 20.0 ± 1.0 nmol with no AEBSF). This small effect showed that AEBSF did not interfere with triggering by phorbol myristate acetate or with the superoxide assay itself.

### Proteomes

For each donor, duplicate 2D gels were run on monocytes treated ± LPS and ± AEBSF. After careful visual inspection to remove debris, bubbles, or speckles of Sypro ruby from the gel images, approximately 1000 protein spots were quantified on each gel by PDQuest software. The density for each spot was normalized against the total spot quantity in the valid protein spots on each gel, using PDQuest. After ANOVA on the means of the spot densities from the duplicate gels from all four donors and all treatments, thirty differentially expressed proteins were found to be statistically significant (P < 0.05) and were studied further. Representative gel images are shown in Additional Files 1, 2, 3 and 4. The identities of the proteins that responded to LPS or AEBSF are shown in Table 1.

**Table 1:** Monocyte proteins altered by LPS or AEBSF. Sorted by Protein ID. Arrows indicate that the protein increased or decreased after LPS or AEBSF

**Behavior**	**Spot**	**Swiss-prot**	**Protein ID**	**MW Gel**	**MW Theory**	**pI Gel**	**pI Theory**
↑AEBSF	K	P07355	Annexin A2	34,000	38,604	8.9	7.57
↓AEBSF	Z	P07355	Annexin A2	29,000	38,604	8.3	7.57
↑LPS	N	P08758	Annexin A5	34,000	35,937	5.2	4.94
↑AEBSF	3Z	P08758	Annexin A5	101,000	35,937	6.4	4.94
↑AEBSF	J	P25705	ATP synthase alpha chain, mitochondrial precursor	50,000	59,751	8.9	9.16
↓LPS	M	P25705	ATP synthase alpha chain, mitochondrial precursor	26,000	59,751	8.6	9.16
↓LPS	V	P00488	Coagulation Factor XIII A chain precursor	90,000	83,267	6.2	5.75
↓AEBSF	Q	P31146	Coronin-1A	50,000	51,026	6.2	6.25
↑LPS	F	P02794	Ferritin heavy chain (N-acetyl)	25,000	21,226	5.7	5.31
↑AEBSF	X	P04406	Glyceraldehyde-3-phosphate dehydrogenase, liver	35,000	36,053	9.2	8.57
↑AEBSF	Y	P04406	Glyceraldehyde-3-phosphate dehydrogenase, liver	35,000	36,053	8.7	8.57
↓LPS	P	P68871	Hemoglobin, subunit beta	10,000	15,998	7.4	6.74
↑LPS	4Z	P22626	HN ribonucleoproteins A2/B1	34,000	37,430	9.3	8.97
↓LPS	T	P08514	Integrin alpha-IIb precursor	130,000	113,391	5.1	5.21
↑LPS	A	P01584	Interleukin-1β precursor	34,000	30,748	4.9	4.70
↑LPS	B	P01584	Interleukin-1β precursor	35,000	30,748	4.8	4.70
↑AEBSF	H	P30740	Leukocyte Elastase Inhibitor	40,000	42,742	6.2	5.90
↓AEBSF	W	P07737	Profilin	12,000	15,054	7.9	8.44
↑AEBSF	C	P14618	Pyruvate Kinase, isozymes M1/M2	57,000	57,937	8.4	7.96
↑AEBSF	I	P14618	Pyruvate Kinase, isozymes M1/M2	60,000	57,937	9.0	7.96
↓AEBSF	R	P14618	Pyruvate Kinase, isozymes M1/M2	36,000	57,937	6.0	7.96
↓AEBSF	5Z	P14618	Pyruvate Kinase, isozymes M1/M2	60,000	57,937	7.0	7.96
↓LPS	2Z	Q15404	Ras suppressor protein 1	30,000	31,540	9.1	8.57
↑LPS	G	P04179	Superoxide dismutase – Mn	25,000	24,722	7.4	8.35
↓AEBSF	L	P29401	Transketolase	57,000	67,878	8.1	7.58
↑AEBSF	U	P29401	Transketolase	70,000	67,898	8.4	7.58
↓LPS	D	P09493	Tropomyosin alpha 1 chain	32,000	32,709	4.9	4.69
↑LPS	O	P08670	Vimentin	50,000	53,652	5.0	5.06
↑LPS	S	P08670	Vimentin	48,000	53,652	4.9	5.06
↓LPS	E	P08670	Vimentin (amino half)	24,000	53,652	4.6	5.06

The pattern of response fell into 4 categories:

1) Increased expression after LPS, with the increase blocked by AEBSF, shown as "↑LPS" in Table 1.

2) Decreased expression after LPS, with the decrease blocked by AEBSF: "↓LPS"

3) Spot absent or minimal after treatment with AEBSF ± LPS: "↓AEBSF"

4) Spot present only after treatment with AEBSF ± LPS: "↑AEBSF"

**Figure 3:**
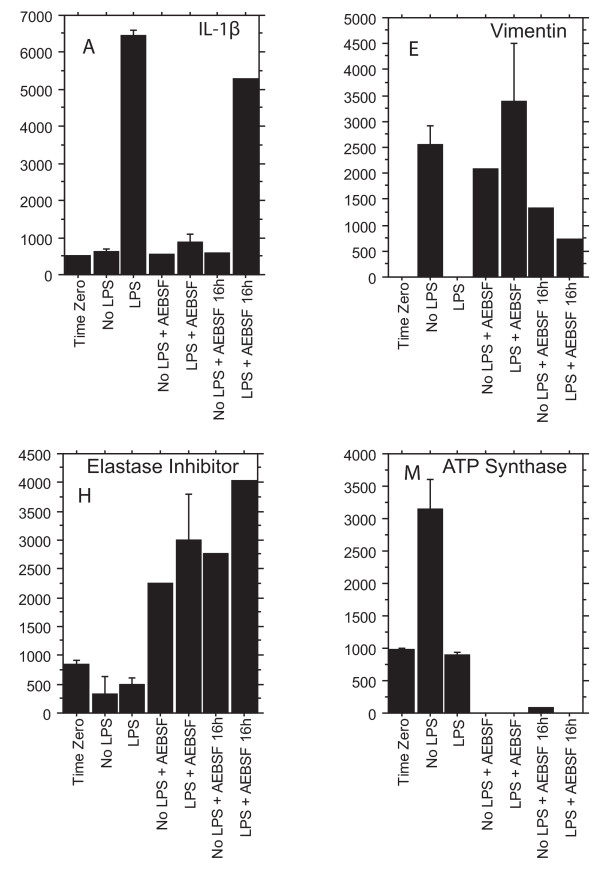
**Effects of LPS and AEBSF on expression of IL-1β, elastase inhibitor, vimentin, and ATP synthase**. Protein levels from 2D gels from monocytes cultured for 16 h ± LPS ± AEBSF are shown for four spots on the gels. The Y-axis represents integrated normalized spot intensity, as calculated by PDQuest. The first bar is Time 0, a control of monocyte cell protein taken at the start of culture. The last two bars show the effect of AEBSF added at the end of the culture at 16 h. Letters designate particular spots on the gel. Results are means ± SE from the last gel run involving the last two donors; results from the first two donors were similar. LPS increased the expression of the particular form of IL-1β precursor found in spot A, but decreased the vimentin spot E and the ATP synthase spot M. AEBSF enhanced expression of elastase inhibitor in spot H. Results from all 30 differing spots are provided in Additional File 5. Representative gels ± LPS ± AEBSF, with spots identified by letter, are shown in Additional Files 1, 2, 3 and 4.

An example of each behavior is shown in Fig 3. Logically, other behaviors were possible, but we did not observe them.

Among the 30 differentially expressed protein spots on the gels, we identified 19 different proteins whose expression changed in response to LPS or AEBSF (Table 1). (Although all 30 spots were identified by mass spectrometry, some of the spots represented different forms of the same protein.)

In response to LPS, IL-1β precursors appeared in the 2D gel (Fig. 3 and Additional File 5, spots A and B). IL-1β precursors decreased after treatment with AEBSF (Fig. 3 and File 5). This observation was surprising, because AEBSF did not affect the secretion of mature IL-1β, as measured by the multiplex immunoassay (Fig. 1). Perhaps AEBSF interfered with the formation of this particular precursor form, but did not block alternative pathways to mature IL-1β. When AEBSF was added at the end of the 16 h incubation, it had no effect (Fig. 3). The observed molecular weights of spots A and B on the gels corresponded to a precursor form of IL-1β (34–35 kDa, compared with 17 kDa for the secreted form). Ion trap MS/MS also showed that spots A and B contained the precursor form of IL-1β. IL-1β has a propeptide of 116 amino acids. For spot A, we identified a peptide ISDHHYSK (11 of 14 possible b and y ions) from the precursor amino-terminus of IL-1β (amino acids 52–59). In spot B, we identified ISDHHYSK and also QAASVVVAMDK (63–73). In both A and B, we identified SLVMSGPYELK (133–143) (15 of 20 ions), which is part of the secreted protein. In spot A, we also found DDKPTLQLESVDPK (191–204) (18 of 26 ions) and FVFNIEINNK (215–225) (16 of 20 ions).

Another interesting finding was that AEBSF increased leukocyte elastase inhibitor (spot H) (Fig. 3, Table 1 and Additional File 5). This protein was definitively identified by ion trap MS/MS in two independent experiments from two donors. Spot H appears to contain the intact leukocyte elastase inhibitor. The molecular weight estimate from the gel was 40 kDa, compared with a theoretical mass of 42,742 Da with 379 amino acids. We have ion trap sequence data for 5 independent peptides: TYNFLPEFLVSTQK (97–110) (21 of 26 b and y ions), EATTNAPFR (178–186) (14 of 16 ions), LGVQDLFNSSK (291–301) (17 of 20 ions), ADLSGMSGAR (302–311) (14 of 18 ions), and DIFISK (312–317) (8 of 10 ions). We also have MALDI-TOF data for: FQSLNADINKR (70–80), TYNFLPEFLVSTQK (97–110), TYGADLASVDFQHASEDAR (111–129), LVLVNAIYFK (159–168), EATTNAPFR (178–186), FKLEESYTLNSDLAR (276–290), HNSSGSILFLGR (364–375). If we permitted phosphorylation in the MALDI peptide search, we added: FALDLFLALSENNPAGNIFISPFSISSAMAMVFLGTR (11–47), HNSSGSILFLGRFSSP (364–379), and LGVQDLFNSSK (291–301). These peptides fit into the sequence range from amino acids 11 to 379. So, in Spot H, we could be missing some of the amino-terminus. We did not detect the peptide containing the putative active site. According to the UniProtKB/Swiss Prot data base entry P30740, the active site of leukocyte elastase inhibitor is amino acids 344–345. The data provide an indication, but not proof, that elastase inhibitor in spot H might be phosphorylated.

AEBSF blocked the increase in superoxide dismutase-Mn associated with LPS priming (spot G) (Table 1 and Additional File 5). AEBSF also blocked priming for phorbol-triggered superoxide release, suggesting that superoxide dismutase appeared in response to, or in anticipation of, oxygen radical stress. AEBSF also eliminated ferritin (spot F) that appeared in response to LPS (Table 1 and Additional File 5).

After identifying the proteins, we inferred that several proteins changed position in the gel after LPS or AEBSF. For example, vimentin appeared to move position in the 2D gel in response to LPS priming. Vimentin, the intermediate filament protein, plays a role in macrophage differentiation [18]. Our results suggested that vimentin was increased by LPS priming (spots O and S at 48–50 kDa on the gels), and that in the absence of LPS, vimentin was cleaved into smaller fragments (spot E at 24 kDa on the gels) (Table 1 and Additional File 5). In contrast, ATP synthase appeared to be cleaved in response to LPS, and this cleavage was blocked by AEBSF (compare results for spot J at 50 kDa with spot M at 26 kDa) (Table 1 and Additional File 5). AEBSF caused transketolase spot L at 57 kDa to disappear and spot U at 70 kDa to increase on the 2D gels (Table 1 and Additional File 5).

Another example of altered position on the gel is Annexin A5. Exposure of monocytes to AEBSF produced a spot corresponding to molecular weight 101,000 Da on the gel (spot 3Z), compared with the theoretical molecular weight of 35,937. Of course, this might be a misidentification, but we found 9 independent peptides, all of which belonged to annexin 5A. Annexin 5A was also found in spot N at 34 kDa, and this spot was increased by LPS (Table 1 and Additional File 5).

## Discussion

This report supplements our previous paper on the proteomics of monocyte priming [17]. Compared with our previous paper, here we analyzed the monocytes at an earlier time after addition of LPS (16 h versus 48 h) to detect protein changes that occur earlier in the response to LPS. LPS causes profound changes in monocyte cell structure and function over time. Nevertheless, many similar protein changes were found in this report, compared with our earlier paper. In that paper, we discussed the relevance of these similar altered proteins to macrophage activation, including superoxide dismutase, annexins, transketolase, pyruvate kinase, and integrin alpha IIb. However, in the earlier paper, because we allowed the events initiated by LPS to unfold for 48 hours, some of the changes that we found may have been rather distant from the initial critical events of priming. In contrast, the results in Table 1 presented here represent changes that occurred early in the response to LPS. Also, on the technical side, we used a less dense 2D gel (12% versus 13% acrylamide/bis) to better separate higher molecular weight proteins (at the cost of losing some lower MW proteins). We also used Sypro ruby fluorescent stain in place of Coomassie blue stain to increase the sensitivity of detection of less abundant proteins. So our previous paper detected some different changes because the effects of LPS had more time to develop, and we were also able to detect changes in some lower molecular weight proteins. The present study detected some different proteins because of greater sensitivity of gel staining and better resolution of higher molecular weight proteins, and because we focused on earlier responses to LPS. Because of these differences between the two studies, comparing the results reported here with those of our previous paper requires some caution and careful thought. A valid time course would require a much bigger study with consistent methodology.

With these caveats in mind, compared with our previous paper, here we found some interesting new protein alterations. The new protein changes included ATP synthase, coagulation factor XIII, coronin, elastase inhibitor, ferritin, glyceraldehyde-3-phosphate dehydrogenase, HN ribonuclear proteins A2/B1, profilin, ras suppressor protein, tropomyosin, and vimentin. In particular, in response to LPS, we saw intracellular precursors of interleukin-1β, one of the most important cytokines elicited by LPS. This observation demonstrated that proteomic analysis could detect changes relevant to macrophage activation.

Jin and colleagues have presented an excellent proteomic reference map of the proteins of blood monocytes [19]. Most the proteins that we detected appear on their map, and they appear in the corresponding locations. Their list includes proteins that we found in unprimed monocytes, like integrin alpha IIb, a protein that disappears after LPS priming [17]. However, their map does not include a number of other proteins that we found to decrease after LPS priming, like coagulation factor XIII and ras suppressor protein. So it is possible that their monocytes were still primed to some extent by their experimental procedures.

Priming or activation must be closely regulated so that a vigorous response to infection can be mounted, while limiting tissue damage by oxygen radicals and cytokines once the infection is controlled. There is abundant evidence for an important role for phosphorylation cascades in regulating the activation of leukocytes [20]. However, in addition to the activity of kinases and phosphatases, we proposed the hypothesis that priming of monocytes in vitro, and also activation of monocytes in vivo in response to infection or inflammation, depends upon the activation of an endogenous protease. Over many years, we attempted to inhibit LPS-priming of monocytes with a wide range of inhibitors, but found inhibition with only two compounds, both protease inhibitors, the most effective being AEBSF [3], an irreversible serine protease inhibitor.

Because AEBSF was the best inhibitor of priming that we found, we hypothesized that priming depended upon the activation of an endogenous protease. We undertook this proteomic analysis to identify the protease or to identify an endogenous inhibitor that keeps that protease from activating monocytes until they are needed to combat infection. We did not observe any change in abundance for any proteases, nor did we come across any proteases covalently modified by AEBSF. However, we saw that AEBSF added to monocytes in vitro caused an increase in the abundance of an endogenous protease inhibitor, leukocyte elastase inhibitor. We speculate that a smooth continuous synthesis of elastase inhibitor is required to maintain monocytes in an unprimed state. If a sufficient amount of endogenous protease is activated (by phosphorylation, for example, and we found alterations in kinases and their substrates), the elastase inhibitor is consumed, and some of the protease survives and causes activation. Alternatively, phosphorylation or dephosphorylation might inactivate the endogenous inhibitor. (We found that elastase inhibitor in spot H was probably phosphorylated.) So, perhaps, elastase inhibitor is a negative regulator of activation. AEBSF might prevent the destruction of this endogenous inhibitor that normally takes place in response to priming by LPS or other priming agents. (Of course, logically, increasing the elastase inhibitor might be a second effect of AEBSF, unrelated to the ability of AEBSF to inhibit priming.) The endogenous leukocyte elastase inhibitor inhibits both serine and cysteine proteases, unlike AEBSF which is specific for serine proteases. If elastase inhibitor is responsible for blocking priming for enhanced release of superoxide, the endogenous protease that might be involved in regulating priming could be either a serine protease or a cysteine protease. However, if AEBSF acts directly on the relevant protease, not just indirectly by sparing elastase inhibitor, then the relevant protease is probably a serine protease.

Regarding the effects of AEBSF on the proteome compared with cytokine secretion, we found that AEBSF had no effect on secretion of IL-1β or the other cytokines, whereas AEBSF blocked the formation of the IL-1β precursors found in spots A and B. We have a direct comparison only with IL-1β, because no other cytokines were detected in the proteome. We suggest that AEBSF inactivated a serine protease that created spots A and B, but that an alternative processing, perhaps involving a non-serine protease, allowed mature IL-1β to be secreted. These apparently conflicting results are surprising, but they were obtained in the same experiments with the same monocyte cultures, assaying simultaneously the secreted cytokines in the medium and analyzing the monocyte cellular proteomes.

Although AEBSF did not affect cytokine release, we showed in earlier work that AEBSF or corticosteroids block priming by LPS for enhanced killing of leukemic cells [21], even though killing of leukemic cells does not require oxygen radicals [22]. So the effects of AEBSF on priming extend beyond superoxide.

We have some speculations about some of the other altered proteins. The high apparent mass of spot 3Z at 101 kDa suggests that AEBSF caused annexin 5A to become conjugated with itself or possibly with another unidentified protein. As their name suggests, the annexins do bind other proteins and lipids.

AEBSF decreased ATP synthase in spot M at 26 kDa and increased ATP synthase in spot J at 50 kDa, which is closer to the theoretical mass of 59,751 Da, suggesting that AEBSF blocked ATP synthase degradation. Besides its role in oxidative phosphorylation, ATP synthase might have an unknown direct role in priming. Because there are only about 25,000 human genes [23], many proteins have multiple functions. For example, we now know that mitochondrial cytochrome c, long studied as part of the electron transport system, is also a key regulator of apoptosis in mouse macrophages [24,25]. It would not be surprising if ATP synthase, another mitochrondrial enzyme, also has a direct role in responses to LPS.

In our study, LPS caused the disappearance of Factor XIII (spot V). Coagulation Factor XIII is a transglutaminase that crosslinks fibrin. As a class, transglutaminases have many functions, and so Factor XIII might have other intracellular roles besides its well known role in stabilizing blood clots. There must also be a function for this protein inside monocytes.

Ras suppressor protein 1 blocks the ras signal transduction pathway [26]. LPS increases active ras-GTP in macrophages [27]. Disappearance of the suppressor protein in response to LPS (spot 2Z) suggests that LPS activated the ras pathway.

This study is far from the last word on the proteomics of macrophage activation. A more extensive time course, from 1 minute to 48 hours after addition of LPS, is needed; and this time course study should utilize a consistent state-of-the-art methodology. Methods other than 2D gels should be attempted. Priming agents other than LPS, like muramyl dipeptide and interferon gamma, should also be examined over time. Other models of macrophage activation should be studied, including the effects of *in vivo *activation of macrophages. Because of the importance of macrophage activation in resistance to infection and in killing cancer cells, these more extensive studies are warranted.

The utility of protease inhibitors like AEBSF as anti-inflammatory drugs remains unknown. Their potential benefits might be overshadowed by side-effects and the risks involved in inhibiting useful host-defence processes. However, it is noteworthy that AEBSF is well-tolerated in animal models [28].

Infectious diseases remain a major problem, due to aging of the population, increased numbers of immuno-compromised patients, and increasing resistance of microbes to antibiotics. Thus, it is important to boost the innate infection-fighting abilities of our immune system. In cell culture and in animal models, we demonstrated that agents that activate macrophages enhance resistance to infection, and that protease inhibitors can block activation. On the other hand, excessive or prolonged macrophage activation, in autoimmune diseases for example, leads to tissue destruction. To correct defects in macrophage activation in human patients we need to know more about the mechanisms that control macrophage activation.

## Conclusion

In a carefully constructed in vitro model of macrophage activation, proteomic analysis revealed that monocytes primed with LPS accumulated intracellular precursors of the key cytokine interleukin-1β. When the priming for enhanced release of oxygen radicals like superoxide was inhibited by AEBSF, an increase in endogenous elastase inhibitor was found. The increase in the elastase inhibitor is consistent with a role for proteolysis in the mechanism of macrophage activation.

## Methods

### Monocytes

Blood was drawn from 4 healthy adult donors at Lifeblood Regional Blood Center in Memphis. The protocol was approved by the University of Tennessee Human Subjects Institutional Review Board. The leukocyte-rich "buffy coats" were isolated by centrifugation, and immediately brought to our laboratory. Monocytes were passively isolated from the buffy coats by RosetteSep (Stem Cell Technologies, Vancouver, Canada), a passive selection technique that does not activate the monocytes. This procedure removed all leukocytes other than monocytes from the cell suspension by crosslinking them to red cells, leaving the monocytes undisturbed. After incubation with the RosetteSep crosslinking antibodies, the cell suspension was layered over Histopaque 1077 (Sigma) and centrifuged, so that red cells and crosslinked white cells pelleted, and the monocytes remained in suspension. EDTA (1 mM) was the anti-coagulant. Cells were counted with a hemocytometer, and the purity of the monocyte population was assessed by microscopic examination and by esterase staining, and found to be >95%. Monocytes were cultured 16 hours overnight ± LPS (2 ng/ml from *E. coli *K12) ± AEBSF (250 μM) in modified Earle's balanced salt solution (EBSS) at 37°C in 5% CO_2 _in Teflon bags. The LPS was given by Floyd C. McIntire of the University of Colorado. AEBSF was purchased from Calbiochem EMD Biosciences Inc., San Diego, CA. Each culture contained 2 million monocytes in 2 ml of medium, corresponding to 200 μg of protein (Lowry method). All reagents used in isolation and culture were endotoxin-free by *Limulus *assay. Solutions were prepared with sterile pyrogen-free bottled "water for irrigation" (Baxter Healthcare, Deerfield, IL, USA). Before use, Teflon bags were baked at 200° for 2 h to destroy any microbial contaminants. Passive separation, absence of contamination, and Teflon are all important to prevent inadvertent activation of monocytes. Multiple cultures were prepared for each experimental condition from each of the donors; one culture was used for confirmation of priming by measuring cytokines and another for measurement of superoxide release, and two cultures were used for duplicate samples for 2D gels. Monocytes from each donor were isolated and cultured in separate experiments performed on different days.

### Cytokines

Monocyte culture media were assayed for 10 cytokines by Multiplex Bead Immunoassay (using a Luminex reader, Bio-Rad, Hercules, CA, USA, and an assay kit from BioSource-Invitrogen, Carlsbad, CA, USA). If monocytes are cultured in endotoxin-free non-adherent conditions, they will produce detectable levels of inflammatory cytokines only after exposure to LPS. By measuring the fluorescence intensity of beads of various infrared "colors", with each color having a cytokine-specific antibody, cytokines like IL-1β, IL-6, IL-8, TNFα, and GM-CSF in the culture media from the four treatment groups (± LPS ± AEBSF) and from multiple donors were quantified simultaneously in the same assay.

### Superoxide

The ability of monocytes to produce superoxide was determined as a further measure of monocyte priming. Superoxide was assayed spectrophotometrically. Sample monocyte cultures were treated with cytochrome c, which changes color from orange to pink when cytochrome c is reduced by superoxide. Superoxide production was triggered in the monocytes by addition of phorbol myristate acetate (1 μM), a phytotoxin that directly activates protein kinase C and triggers superoxide release. Using a Cary 300 spectrophotometer (Varian, Palo Alto, CA, USA), the height of the absorbance peak of reduced cytochrome c was measured at 550 nm, and compared with isosbestic points at 542 nm and 556 nm (wavelengths at which the absorbance does not change during oxidation-reduction). Because cytochrome c has a narrow absorbance peak, this analysis required a spectrophotometer with wavelength accuracy of 0.5 nm and wavelength resolution of 0.5 nm. The extinction coefficient for reduced versus oxidized cytochrome c of 0.021 μM^-1 ^was used to calculate nanomoles of superoxide released per million monocytes.

### 2D gels

Monocytes from each of the cultures in Teflon bags were washed with saline, and pelleted by centrifugation. The monocytes in the pellet were digested with 0.1 ml of a solution containing urea (7 M), thiourea (2 M), and CHAPS detergent (4%). Using a Reduction-Alkylation kit (Bio-Rad, Hercules, CA, USA), the protein disulfide bonds were reduced with tributylphosphine, and alkylated with iodoacetamide. The samples were then treated with a 2D Clean-Up kit (Bio-Rad), which precipitated the proteins with organic solvents, removing small molecules. These kits were essential for good resolution in the pH 8–10 range on the 2D gels. The cleaned-up protein pellets were dissolved in 400 μl of "rehydration buffer". Rehydration buffer contained urea (7 M), thiourea (2 M), CHAPS (4%), 30 μl of 100× ampholytes pH 3–10 (Bio-Rad), and bottled water-for-irrigation in a final volume of 3 ml.

The cellular proteins were separated using large-format 2D gels (20 cm × 20 cm × 1 mm), with a pH range of 3–10, and a useful molecular weight range of 10–250 kDa. For the first dimension, 350 μl of each sample was pipetted into a 12 slot tray. Isoelectric focusing strips (ReadyStrip, 17 mm, pH 3–10, linear, Bio-Rad) were placed face down onto the samples, and the strips were allowed to rehydrate and absorb the samples overnight. Care was taken to ensure that the strips did not adhere to the trays, which might interfere with even rehydration. The tray was wrapped in plastic with a slightly damp paper towel included to prevent evaporation of the samples. The first dimension was run in a Bio-Rad Protean IEF cell. Samples were subjected to isoelectric focusing for a total of more than 80,000 kVh, with a slow ramp-up of voltage 200 V to 10,000 V over 1 h, then 10,000 V for 8 h. The focused strips were then subjected to a second round of reduction and alkylation, using dithiothreitol (130 mM) and iodoacetamide (135 mM), for 10 min in each solution, with 3 ml of each solution in each slot of a 12 slot tray.

For the second dimension of SDS-PAGE, 2D gels were run 12 at a time using the Bio-Rad Protean plus Dodeca system. The Dodeca cell for the second dimension was critical for producing gels that were sufficiently reproducible to allow the PDQuest software to make objective comparisons of spot intensities without significant human intervention or bias. We cast 12% gels, containing 198 ml of 40% acrylamide/bis (29:1), 165 ml of Tris-HCl, pH 8.8, and 297 ml of bottled water, for a total volume of 660 ml. The solution was degassed with a vacuum pump for 5 min, 3.3 ml of 10% ammonium persulfate and 165 μl of TEMED were added and gently swirled into the mixture. The gel mixture was added to the casting chamber, and the cassettes were filled from the bottom. Water was added to the top of the gels with a 12-tip pipetter to prevent "waves" as the gels polymerized. Running buffer was added to the Dodeca unit, and maintained at 15–20° with a high capacity (>400 watts) refrigerated circulator. Running buffer (25 liters) contained Tris base (25 mM), glycine (192 mM), and sodium dodecyl sulfate (0.1%) (pH 8.3). The focused strips were dipped in 2D gel running buffer. The damp strip was placed on the glass near the top of the gel in the cassette, 5 ml of hot agarose (0.5%) was added to the top of the gel, and the strips were pushed down into the agarose. On either side of the strip, filter paper squares (2 mm × 2 mm) containing 2 μl of protein standards were also pushed into the agarose before it hardened. The cassettes were placed sideways into the Dodeca apparatus, where electrophoresis moved proteins from the right to the left. Electrophoresis was conducted at 200 V for 400 min.

Gels were removed from the cassettes and allowed to fall gently into 250 ml of 10% methanol, 7% acetic acid in plastic trays. Gels were rocked gently for 30 min. The gels were stained for 4 hours with Sypro ruby, washed for 30 min with 10% methanol, 7% acetic acid, and washed finally in water, and stored in the refrigerator. The wet gels were scanned on a BioRad FX fluorescence scanner. Gels from each of the donors were analyzed and compared using PDQuest software. The results from all four donors were later compared and analyzed by ANOVA (see Statistical analysis below).

The protein spots were manually cut from the wet gels, using a UV light box to visualize the orange-red fluorescent spots, and using pipette tips, cut to the appropriate diameter, as punches. The gel plugs were washed with water, and incubated in ammonium bicarbonate (200 mM, pH 8.5) for 20 min. After the incubation, the gel plugs were dehydrated with acetonitrile, and were dried in a vacuum centrifuge. Each dried gel plug was rehydrated with 20 μl of 50 mM ammonium bicarbonate containing sequencing-grade trypsin (Promega, Madison, WI, USA) at a concentration of 16.7 ng/μl. Digestion was carried out overnight at 37°C. The trypsin-digested samples were centrifuged, and the supernatants were collected and placed in siliconized Eppendorf test tubes (Fisher Scientific, Pittsburgh, PA, USA). To extract residual peptides, a solution of 35% water/60% acetonitrile/5% trifluoroacetic acid was added to the remaining gel plugs, the samples were subjected to an ultrasonic water bath for 20 min, and centrifuged (12,000 × g for 1 min). Following centrifugation, the supernatants were recovered, and were combined with the previously collected supernatants. The tryptic peptides were dried in a vacuum centrifuge. Before the nanoLC-MS/MS analysis, each digest was purified with a ZipTip C18 microcolumn (Millipore, Bedford, MA, USA). The resulting peptides were eluted from the ZipTip with 3 μl of 50% water/50% acetonitrile/0.1% trifluoroacetic acid. After elution, each sample was diluted with 3 μl of water/0.1% trifluoroacetic acid.

### Mass spectrometry

Proteins were identified by liquid chromatography-nanoelectrospray ionization quadrupole ion trap mass spectrometry, using an LCQ Deca or an LCQ Deca XP Plus mass spectrometer (ThermoElectron, San Jose, CA, USA) to obtain MS and MS/MS spectra. Immediately following the ZipTip purification, samples were manually injected through a 6-port NanoPeak valve (M-485) from Upchurch Scientific (Oak Harbor, WA, USA), fitted with a 2 μl capillary PEEK (polyether ether ketone plastic) sample loop. The samples were loaded onto a 75 μm I.D. (360 μm O.D., 15 μm tip, 10.5 cm length) Picotip capillary column/spray needle (New Objective, Woburn, MA, USA) packed in-house with MAGIC C18 reversed-phase material (5 μm, 200 Å) from Michrom Bioresources (Auburn, CA, USA). Chromatography started with a 5 min initial isocratic step of 100% A, followed by a linear 0–90% B gradient for 40 min, followed by 25 min of 90% B. (A = water with 2% methanol and 0.1% formic acid; B = 90% methanol, 10% water and 0.1% formic acid). Peptides were eluted with a 200 nl/min flowrate. Spectra were acquired with the instrument operating in the data-dependent mode. The instrument cycled through the acquisition of a full-scan mass spectrum, followed by MS/MS scans of the five most prominent ions after collision with helium gas. Results were analysed with SEQUEST software (Sequest Technologies, Lisle, IL), which matched observed peptide masses and product ion masses with the theoretical values for all proteins from humans in the UniProt/Swiss Protein database [29]. A maximum of one missed cleavage was allowed, and the isotopic resolution of the experimental masses was specified as monoisotopic. The experimental pI and MW from the gels were not used to restrict the SEQUEST search. Modifications specified in the search were that cysteine was carbamidomethylated, and that methionine could be oxidized. Identification required an Xcorr ≥ 2.50 for doubly charged ions for at least two independent peptides.

As a supplement to ion trap mass spectrometers, Matrix Assisted Laser Desorption/Ionization Time-of-Flight (MALDI-TOF) mass spectrometers were used for peptide mass fingerprinting. The purified tryptic peptide samples were mixed with a matrix of α-cyano-4-hydroxycinnamic acid, spotted on a metal plate coated with paraffin wax [30], allowed to dry, and washed. The plate was loaded into a Voyager DE-RP MALDI-TOF (Applied Biosystems, Foster City, CA) or a Bruker Ultraflex MALDI-TOF/TOF mass spectrometer (Bruker Daltronics, Billerica, MA). The mass-to-charge ratio (m/z) was determined for each peptide (in these conditions, z = 1). The masses of the tryptic peptides were determined from the mass spectrum produced, and compared with the theoretical masses of tryptic peptides from all known human proteins to establish the identity of the proteins of interest. MS-Fit [31] or Mascot [32] and the UniProt/SwissProt database [29] were used for protein identification. Masses were monoisotopic, cysteines were carbamidomethylated, and one missed cleavage was allowed. Possible modifications considered were oxidation of M, N-terminal gln to pyroglu, N-terminus acetylated, and phosphorylation of S, T, or Y. (The second time-of-flight capability of the Bruker instrument, which determines the mass of fragments of the tryptic peptides, was not used for these experiments.)

### Statistical analysis

From each donor and each treatment, monocyte protein digests were prepared and run on duplicate gels. The images of each gel were compared with PDQuest software, which determined the densities (volume by Gaussian integration) of each of the protein spots on the gels. The two gels from the same sample gave spot densities that differed only slightly (standard deviation < 10%). Any greater deviation was an indication of a spot mismatch, which was corrected. For each donor, the PDQuest analysis was then used to examine whether any of the spots changed in density as a result of treatment ± LPS ± AEBSF. The means of the spot densities of the spots that changed in all 4 donors were then subjected to analysis of variance (ANOVA) followed by Scheffe's F-test to determine whether any individual differences in protein expression ± LPS ± AEBSF were significant at P < 0.05 level, with N = 4. Each of the 30 proteins listed in Table 1 showed significant differences by this analysis. (ANOVA corrects for the problem of the large number of protein spots being analysed, so that 1 in 20 results (P < 0.05) are *not *assigned significance by random chance.) Two of the donors did not yield sufficient monocytes to allow for the particular controls involving AEBSF added only after the 16 h incubation ± LPS, so N = 2 for those particular controls. Although those controls were included in the overall ANOVA analysis, the results from those controls should not be overly relied upon. Of course, statistical significance does not mean biological importance. In the Discussion here and in our previous paper [17], we try to evaluate the biological importance of the statistically significant changes that we observed.

## Competing interests

The authors declare that they have no competing interests.

## Authors' contributions

The study was designed by MJP; executed by MJP, KMP, and DBH; and the mass spectrometry was performed and interpreted by SBG and FG. All authors read and approved the final manuscript.

## Additional files

The four 2D gel image files (Additional Files 1, 2, 3 and 4) should be viewed on a high quality large monitor, using 200–600% magnification as provided by Acrobat Reader. A print-out on paper fails to convey the resolution or bit-depth of the images and over-emphasizes streaking. The original scanned images from the FX fluorescence scanner have thousands of levels of fluorescence and 3.1 million pixels, and these were the images actually used in the PDQuest analysis.

## Supplementary Material

Additional File 1 **2D gel image from unprimed monocytes (No LPS)**. 2D gel from monocytes incubated for 16 h with no addition. Stained with Sypro ruby. Spots (or the positions of missing spots) are located just to the left of the identifying letters. Click here for file

Additional File 2 **2D gel image from monocytes primed with LPS**. 2D gel from monocytes exposed to LPS (2 ng/ml) for 16 h. Click here for file

Additional File 3 **2D gel image from unprimed monocytes treated with AEBSF**. 2D gel from monocytes exposed to AEBSF (250 μM) for 16 h in the absence of LPS. Click here for file

Additional File 4 **2D gel image from monocytes primed with LPS and treated with AEBSF**. 2D gel from monocytes exposed to AEBSF (250 μM) for 16 h in the presence of LPS. Click here for file

Additional File 5 **Histograms of 30 differentially expressed protein spots in 2D gels from monocytes treated ± LPS ± AEBSF**. The first bar is Time 0, a control from monocytes taken at the start of culture. The second bar is No LPS for 16 h, third bar is LPS for 16 h, fourth bar is AEBSF alone for 16 h, and fifth bar is LPS + AEBSF for 16 h. The sixth bar shows the effect of AEBSF added at the end of culture at 16 h to monocytes not treated with LPS, and the seventh bar shows AEBSF added at the end of culture to monocytes exposed to LPS for 16 h. Four patterns of expression were observed: yellow background indicates proteins whose expression increased in response to AEBSF, pink indicates proteins that decreased with AEBSF, blue indicates proteins that decreased with LPS, and green indicates proteins that increased with LPS. Click here for file
