# Supplementary material for: Proteome of monocyte priming by lipopolysaccharide, including changes in interleukin-1beta and leukocyte elastase inhibitor
Source: Proteome Sci. 2008 May 20;6:13. doi: 10.1186/1477-5956-6-13 (PMC2413206; doi:10.1186/1477-5956-6-13)
Supplement: Additional File 1 — 2D gel image from unprimed monocytes (No LPS). 2D gel from monocytes incubated for 16 h with no addition. Stained with Sypro ruby. Spots (or the positions of missing spots) are located just to the left of the identifying letters. [file 1477-5956-6-13-S1.pdf]

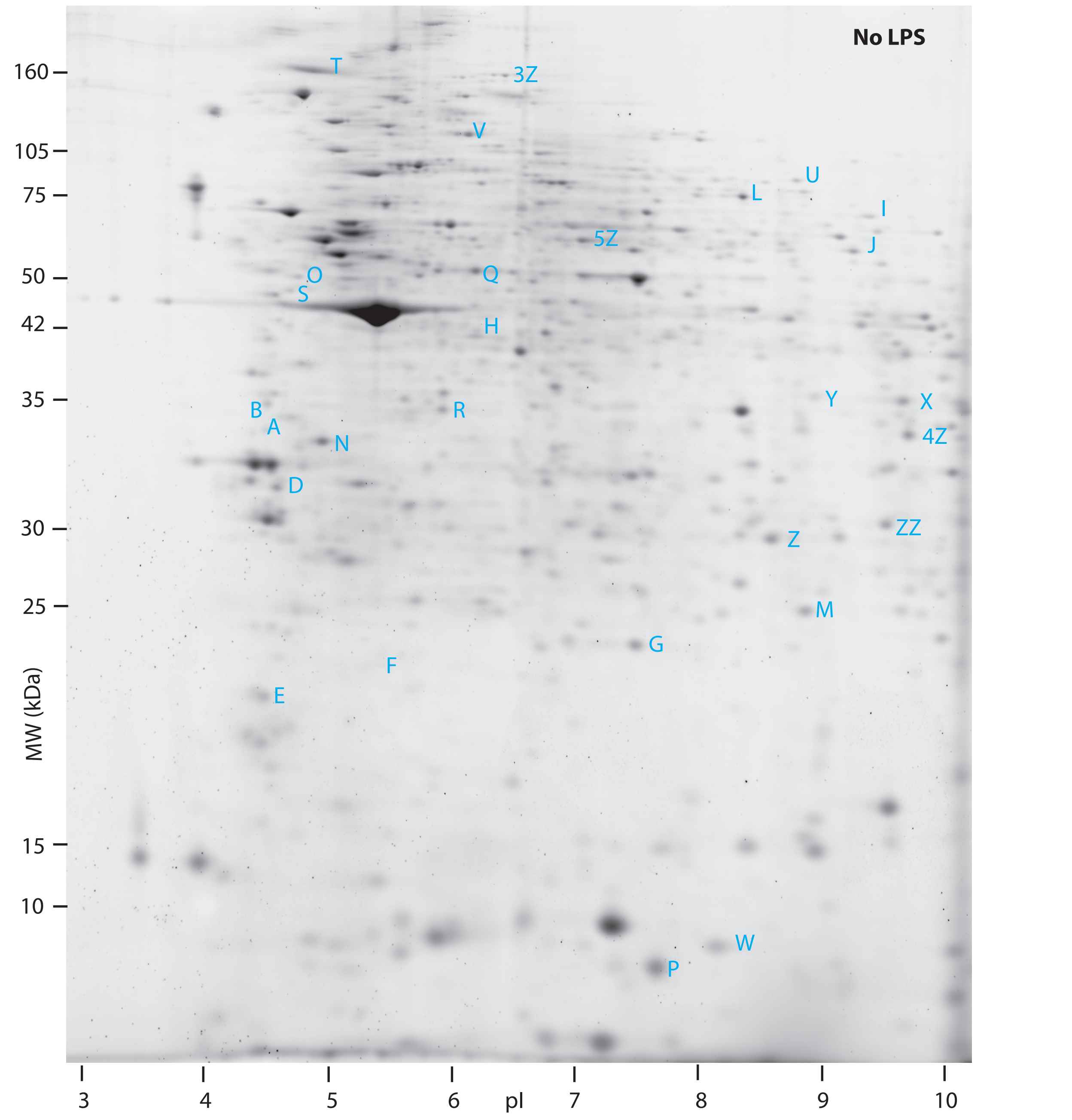
No LPS
MW (kDa)
160
105
75
50
42
35
30
25
15
10
pI
3
4
5
6
7
8
9
10
T
3Z
V
U
L
I
J
5Z
O
S
Q
H
B
A
N
R
D
Y
X
4Z
ZZ
Z
M
G
F
E
W
P
