# Supplementary figures and images for: Proteome of monocyte priming by lipopolysaccharide, including changes in interleukin-1beta and leukocyte elastase inhibitor
Source: Proteome Sci. 2008 May 20;6:13. doi: 10.1186/1477-5956-6-13 (PMC2413206; doi:10.1186/1477-5956-6-13)

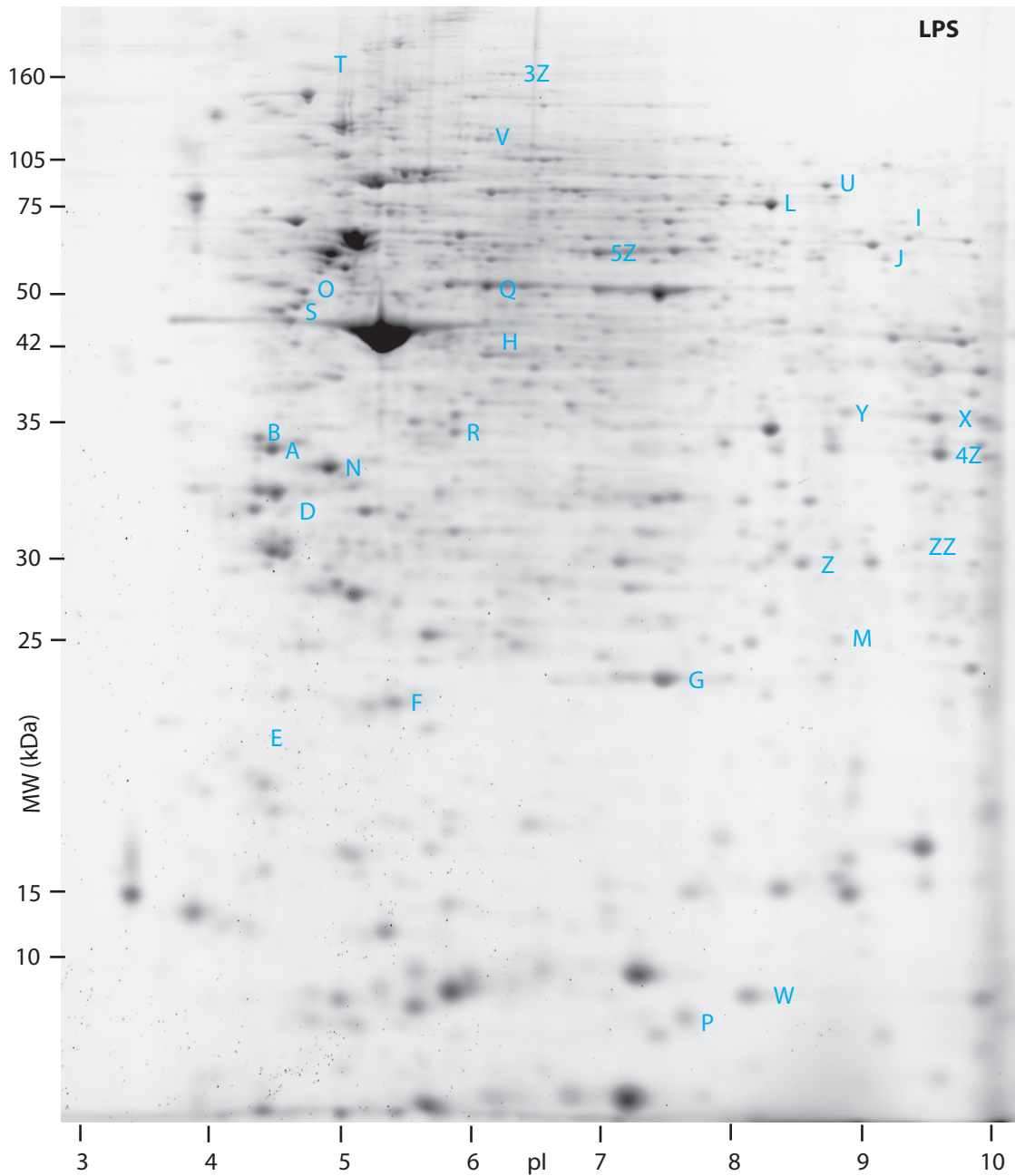

Supplement: Additional File 2 — 2D gel image from monocytes primed with LPS. 2D gel from monocytes exposed to LPS (2 ng/ml) for 16 h. [file 1477-5956-6-13-S2.pdf]

No LPS + AEBSF

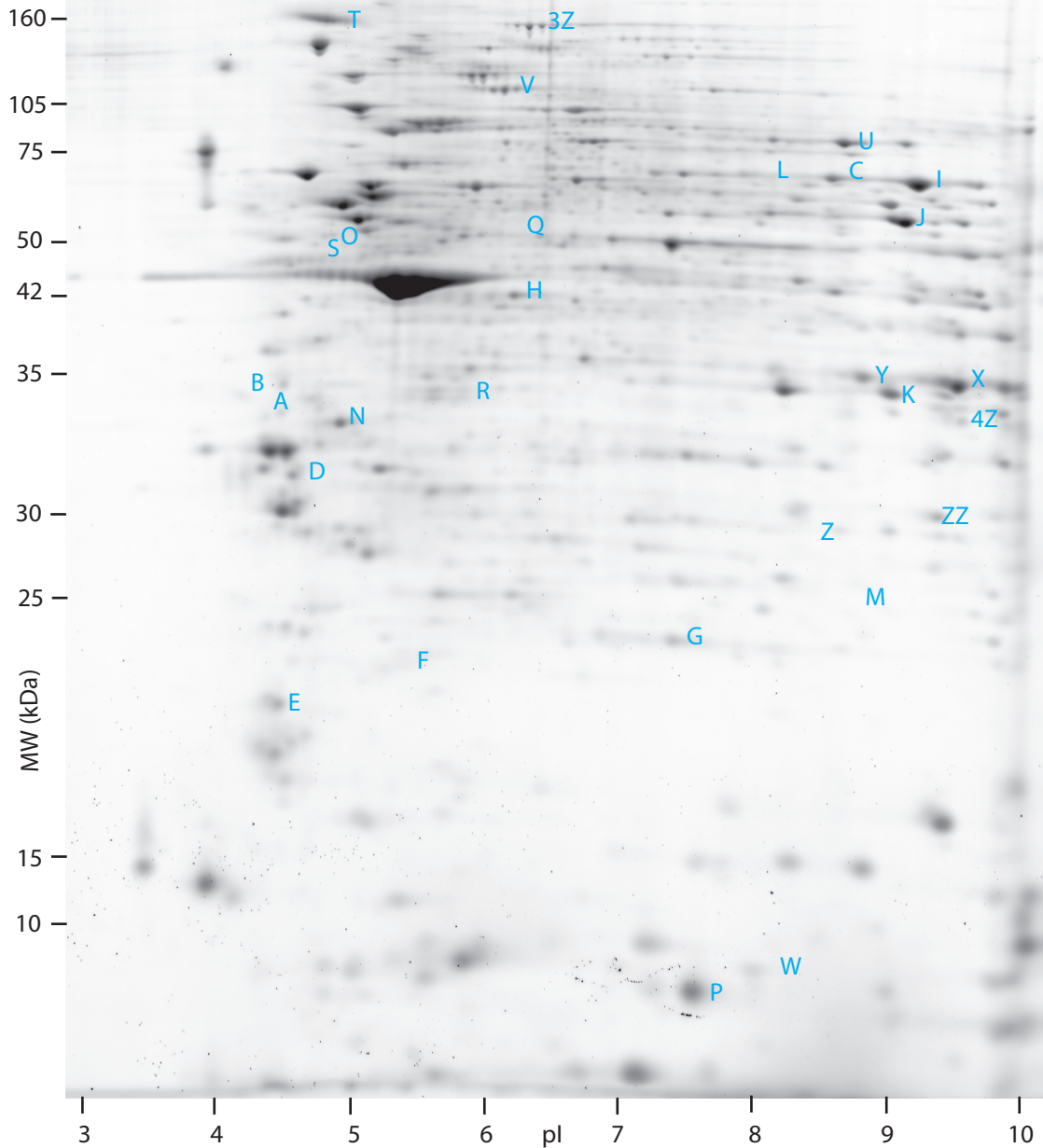

Supplement: Additional File 3 — 2D gel image from unprimed monocytes treated with AEBSF. 2D gel from monocytes exposed to AEBSF (250 μM) for 16 h in the absence of LPS. [file 1477-5956-6-13-S3.pdf]

**LPS + AEBSF**

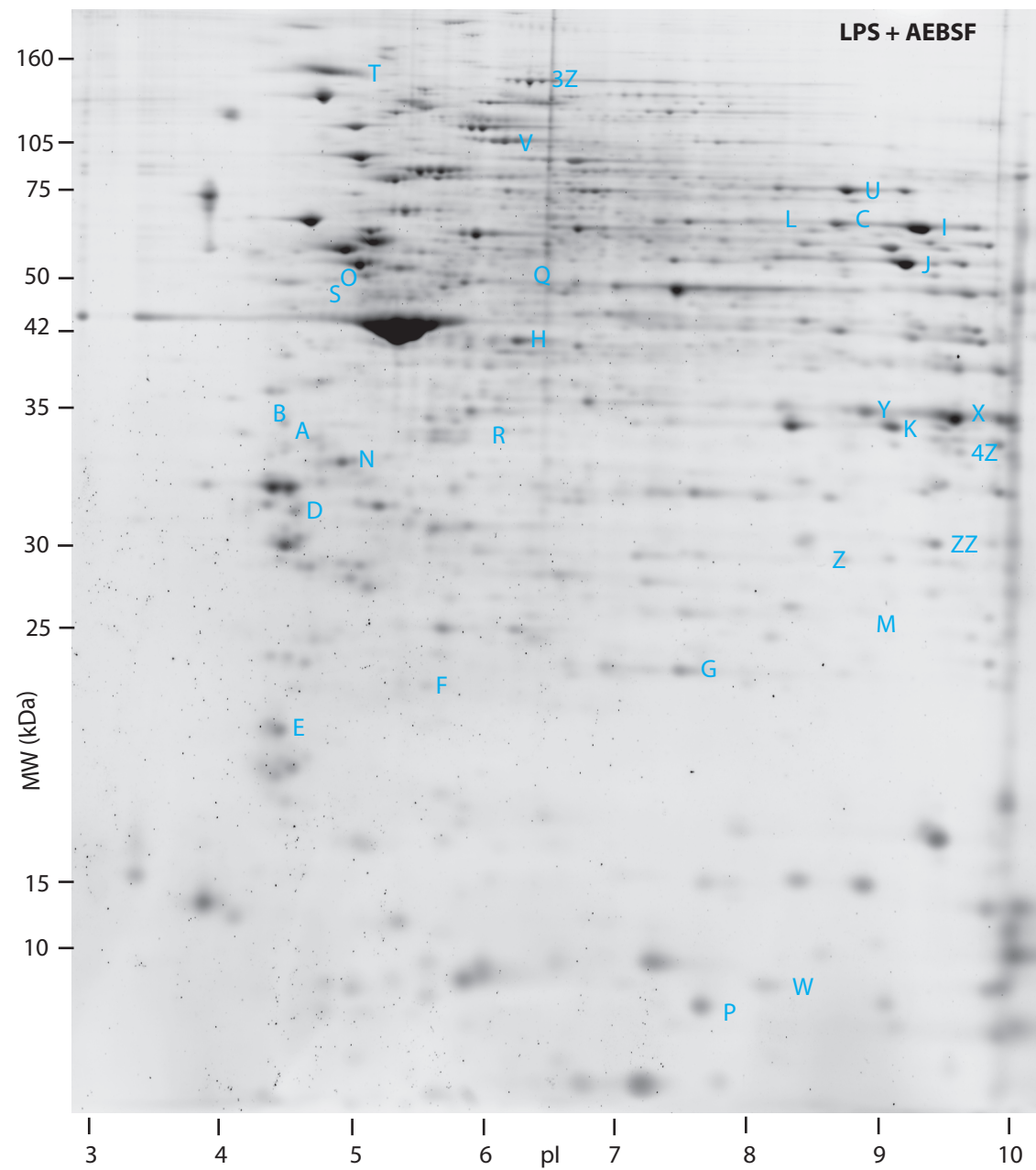

Supplement: Additional File 4 — 2D gel image from monocytes primed with LPS and treated with AEBSF. 2D gel from monocytes exposed to AEBSF (250 μM) for 16 h in the presence of LPS. [file 1477-5956-6-13-S4.pdf]

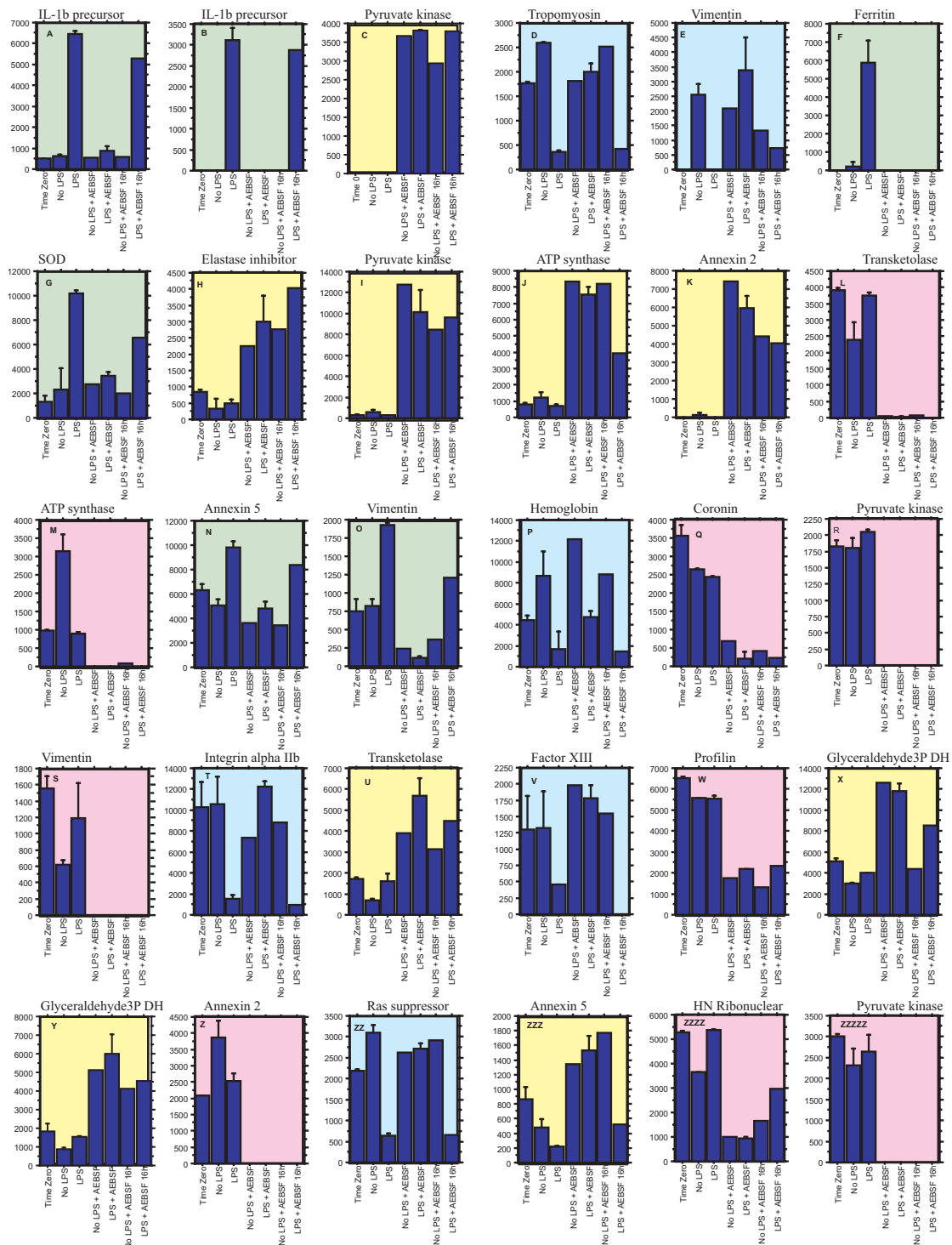

Up with AEBSF
Down with AEBSF
Down with LPS
Up with LPS

Supplement: Additional File 5 — Histograms of 30 differentially expressed protein spots in 2D gels from monocytes treated ± LPS ± AEBSF. The first bar is Time 0, a control from monocytes taken at the start of culture. The second bar is No LPS for 16 h, third bar is LPS for 16 h, fourth bar is AEBSF alone for 16 h, and fifth bar is LPS + AEBSF for 16 h. The sixth bar shows the effect of AEBSF added at the end of culture at 16 h to monocytes not treated with LPS, and the seventh bar shows AEBSF added at the end of culture to monocytes exposed to LPS for 16 h. Four patterns of expression were observed: yellow background indicates proteins whose expression increased in response to AEBSF, pink indicates proteins that decreased with AEBSF, blue indicates proteins that decreased with LPS, and green indicates proteins that increased with LPS. [file 1477-5956-6-13-S5.pdf]
